# The impact of pharmacy benefit managers on community pharmacy: A scoping review

**DOI:** 10.1016/j.rcsop.2023.100283

**Published:** 2023-05-27

**Authors:** Meagen Rosenthal, Lindsey Miller, Mixson Bateman, Megan Smith, Katrina Nueva, Jordan M. Ballou

**Affiliations:** a Department of Pharmacy Administration, University of Mississippi School of Pharmacy, University, MS, United States of America; b Community-based Pharmacy Resident at Moose Pharmacy, The University of North Carolina Eshelman School of Pharmacy in Chapel Hill, NC, United States of America; c PGY-1 Resident, Baptist Memorial Hospital-Memphis, Memphis, TN, United States of America; d Department of Pharmacy Practice, University of Arkansas for Medical Sciences College of Pharmacy, Little Rock, AR, United States of America; e University of Mississippi School of Pharmacy, Jackson, MS, United States of America; f Department of Clinical Pharmacy and Outcomes Sciences, University of South Carolina College of Pharmacy, Columbia, SC, United States of America

**Keywords:** Community pharmacy services, Pharmacy benefit managers, Medication access, Insurance, Pharmaceutical services

## Abstract

**Introduction:**

The introduction of pharmacy benefit managers (PBMs) within the United States healthcare system occurred with the aim to decrease costs and increase quality. News media and legislation have painted a picture of decreased pharmacy competition and potential negative impacts on patients and their access to affordable medications.

**Objective:**

The objective of this scoping review was to evaluate the current research literature examining the impact of PBMs on the finances of community pharmacies.

**Methods:**

Scientific journal articles published between 2010 and 2022 were included if they met the predefined objective.

**Results:**

This scoping review identified four articles that met inclusion criteria. None of the identified articles independently quantified the financial impact of PBMs on community pharmacies.

**Conclusions:**

Additional research should be completed to specifically understand the financial impact on community pharmacies to help ensure the viability of community pharmacy as an integral access point for patients.

## 1 Introduction

Despite the identification of the limited availability of healthcare resources over twenty years ago, healthcare shortages and difficulty in obtaining access to primary care in rural areas of the United States (US) persist.^1^, ^2^, ^3^, ^4^ An especially important component of primary care is affordability of medications to maintain health.^5^ However, the public often does not see the complex system that determines the price of prescription medications. Historically, a pharmacy would submit an insurance claim for a prescription, which would include the cost of the medication and a dispensing fee. The insurance company would then reimburse the pharmacy for that amount if the drug was covered by their plan.^6^ Insurance companies in the US began to see a need to contain their own drug spending in the 1960s, while also minimizing financial burden to the patient.^6^^,^^7^ The outcome of this was the creation of the pharmacy benefit manager (PBM) system.^6^^,^^7^

Often called the “middleman” of the US pharmaceutical industry, PBMs today are third party administrators that exist between community pharmacies and insurance companies, as well as between insurance companies and drug manufacturers.^8^ The PBM system determines formulary coverage, sets copay tiers, processes drug claims, and negotiates contracts between insurers, manufacturers, and dispensing pharmacies.^9^ The main purpose of PBMs is to give consumers affordable access to prescription medication while simultaneously keeping costs at a minimum for a variety of health insurance plans, including Medicare Part D plans, Medicaid managed plans, commercial plans, and self-insured plans.^7^ Given this central location of PBMs in price negotiations and drug plan design, they have the ability to integrate manufacturer discounts into these designs without necessarily revealing them to pharmacies.^10^ This results in potentially lower profits for community pharmacies.^10^

This “middleman” status also means that PBMs are key players in determining patient access to medications.^7^ Manufacturers can provide rebates (partial refunds) to PBMs in return for their medications being placed favorably in the tiered coverage system often utilized by PBMs, where drugs of a lower tier are more likely to be covered by insurance and, therefore, more likely to be prescribed for patients.^7^ PBMs can further incentivize the use of more profitable medications through the use of the same tiered formulary system, and erect barriers to coverage such as the need for prior authorizations and step-therapy requirements.^7^ PBMs have also worked to increase the use of mail order pharmacies through various mechanisms as an additional approach to curbing dispensing costs and passing the savings along to patients.^7^

One of the primary concerns of community pharmacists with the current PBM business model involves the lack of transparency with all the pricing mechanisms mentioned above, with a large focus on direct and indirect remuneration (DIR) fees.^11^ DIR fees were originally intended as a way to track rebates. Currently, the phrase “DIR fees” has become a broad term including any remuneration pharmacies are expected to pay PBMs post-sale.^12^ These price adjustments are not reflected at the point of sale and often result in a negative profit margin for the dispensing pharmacy.^11^ This post-sale price adjustment is often called a “clawback” due to the fact that pharmacies are not able to plan for them and have no recourse once they are requested by the PBM.^13^^,^^14^

Recent discussions surrounding PBMs have raised the question of how much they truly benefit the patient if they don't outweigh the increased out-of-pocket costs that patients pay for prescription drugs.^11^ For example, a recent article focusing on cancer treatments found that, depending on the treatment, patients may be expected to cover between 30% and 50% of drug costs.^15^ Another study found that more than 75% of abandoned prescriptions for a cholesterol treatment medication were due to patient co-pay amounts.^16^

To date, efforts to address these issues for patients has focused on addressing the impact of PBMs at either a policy level or through the court system.^17^^,^^18^ While this work is very important, with tremendous progress being made in this space, additional data are needed. There is anecdotal evidence and many commentaries discussing the impact of PBMs on pharmacy practice as it relates to poor reimbursements, mail order pharmacies, and steering patients away from traditional community pharmacies.^17^ The objective of this scoping review was to evaluate the current literature examining the impact of PBMs on the finances of community pharmacies.

## 2 Methods

The design of this study was a scoping review. Scoping reviews provide an opportunity to observe all the literature currently available with an eye towards generating research questions for future projects. Arksey and O'Malley (2005) provide a five-stage framework which was utilized to guide this scoping review: (1) specify the research question, (2) identify relevant literature, (3) select studies, (4) map out the data, and (5) summarize, synthesize, and report the results.^19^ The research question for this scoping review has been outlined in the background section, and was driven by the desire of community pharmacists and researchers to better understand the current health insurance model of PBMs and how it has impacted the sustainability of community pharmacies in their communities.

Search terms for this scoping review included: community pharmacy, impact, [PBM OR Pharmacy Benefit Managers], DIR fees, [clawbacks OR clawing back], independent, rural, [point of sale OR POS], retroactive fees, third-party payer, middlemen, [PCMA OR Pharmaceutical Care Management Association], price concessions, legislative action, [ERISA OR Employee Retirement Income Security Act of 1974], [MAC or Maximum Allowable Cost], rebates, intermediary, gross revenue, and net revenue. Searches were conducted in three separate databases: Pubmed, IPA, and ProQuest, for literature published within the predetermined time frame. Each database was searched separately, and all articles identified were considered separately.

To be included, an article had to be published between 2010 and 2023 in the US, be available in English, and contain a research question, aim, or objective measuring the financial impact of PBMs on community pharmacy practice. In addition to not meeting one of the inclusion criteria, articles were excluded if they were perspective, commentary, or opinion pieces, or if full text versions could not be located.

The search strategy included filters for the exclusion criteria time frame, so these articles were automatically removed. Upon completion of the searches in each of the database's duplicate articles were removed. Two readers (LM, MB) independently conducted a title review, abstract review, and complete article review. All investigators discussed the potential removal of articles after each review phase to ensure agreement and understanding. Articles were removed if the investigators determined they did not fit the study objective, or if they were editorials, personal letters, or commentaries. After compiling the final list of articles, the data extraction process began and was guided by a table matrix built *a priori* based on work by Arksey and O'Malley (2005).^19^ All studies included after the complete article review were discussed a final time by all investigators. This group discussion confirmed the accuracy and validity of the included articles along with an understanding of the findings in relation to the objective of this scoping review.

## 3 Results

**Fig. 1:**
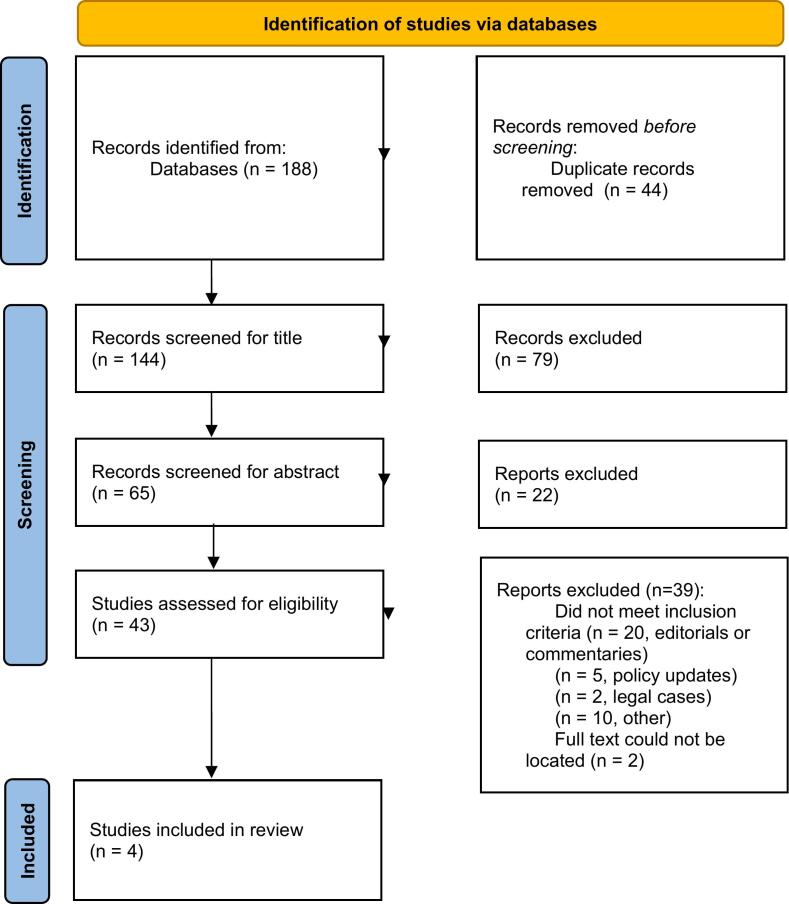
*From:* Page MJ, McKenzie JE, Bossuyt PM, Boutron I, Hoffmann TC, Mulrow CD, et al. The PRISMA 2020 statement: an updated guideline for reporting systematic reviews. BMJ 2021;372:n71. https://doi.org/10.1136/bmj.n71 For more information, visit: http://www.prisma-statement.org/.

Through the keyword searches, 188 articles were identified (see Fig. 1 for PRISMA flow diagram). After removing duplicates, 144 original articles remained. During the title review stage, 79 articles were ruled not eligible, leaving 65 articles. In the abstract review, 22 articles were ruled out, leaving 43 articles. After the full-text reviews, 39 more articles were removed; Full access for two of these articles was unavailable. All removals were based on the fit of the article to the study objective to understand the financial impact of PBMs on community pharmacy practice. This left four full-text articles to be included for complete data extraction and interpretation.

Of the four final articles, one article explored cost savings associated with PBM reimbursement to pharmacies of pharmacist drug therapy modification.^20^ One study was a survey conducted by the National Community Pharmacists Association (NCPA) and focused on better understanding community pharmacists perspectives on PBM fee structures.^21^ One study asked pharmacists about their concerns with the impact of PBMs on their businesses.^22^ One project provided information regarding the National Average Drug Acquisition Cost (NADAC), a new tool designed to improve pricing transparency for community pharmacies.^23^

The primary finding from the reimbursement study was that PBM reimbursement to pharmacies for drug therapy modifications saved $40,927, while costing $9000 for the 750 claims examined.^20^ Moreover, the drug therapy modifications saved patients a total $29,747 for drug therapy changes, tablet splitting, and drugs not covered.^20^ In the survey of 640 community pharmacists, NCPA found most respondents never received an itemized accounting of money deducted from claims (73%), these fees were only assessed quarterly (54%), and pharmacies experienced co-pay “claw-backs” between 10 and 50 times in the previous month.^21^ Additionally, a 2022 study conducted by PioneerRx, a pharmacy software vendor, found that 100% of pharmacies surveyed are concerned about the impact PBMs will have on their businesses, and only 50% are “somewhat familiar” with how PBMs work.^22^ Additionally, 38% of pharmacies surveyed had cut the numbers or hours of their staff, while nearly 30% had reduced the services that they offered, in an effort to combat the increased DIR fees.^22^ The final article outlines a tool (the NADAC) which offers community pharmacists the ability see the national average acquisition cost of drugs plus a professional dispensing fee for all independent retail and community pharmacists, as well as chain pharmacies that provide Medicaid programs.^23^ The website which hosts the data is run by Medicaid, and while not comprehensive, does provide insights that are not otherwise available to pharmacists.^23^^,^^24^

## 4 Discussion

This scoping review identified four articles that met inclusion criteria. These articles covered cost savings associated with PBM reimbursement for drug therapy modifications,^20^ a survey to better understand community pharmacists perspectives on PBM fee structures,^21^ another survey examining the self-reported financial impact of PBMs on community pharmacy practice,^22^ and a project outlining a new tool designed for improving pricing transparency.^23^ Two of the included studies pertained specifically to community pharmacy practice and the self-reported impacts they have faced through the conversion to a PBM-mediated pharmaceutical industry, but failed to measure direct and specific financial impact.^21^^,^^22^ There were no studies to quantify the financial and clinical effects on both pharmacies and patients. The lack of information for how community pharmacies are being affected identifies a large gap in research-based knowledge.

Despite this lack of research-based knowledge about the financial impact of PBMs on community pharmacy practice, there has been a significant amount of discussion in other areas of pharmacy practice. Of the 43 full articles assessed for inclusion in this scoping review, 20 were editorials or commentaries published in community pharmacy focused trade journals, five were policy updates, and two were reviews of legal cases (Fig. 1). Taken together this suggests that there is an appetite for information related to the impact of PBMs despite the apparent lack of published research focused on the impact on community pharmacy practice.

As the number of legal cases, laws, and new bills designed to curtail the traditional functioning of PBMs continues to increase, establishing a solid base of evidence outlining the impact of these traditional practices on patients and community pharmacies is key.^25^ This is particularly important for rural community pharmacy, which faces a unique set of challenges related to patient population, healthcare access, and geographic distance. Without this evidence base, it is possible, and potentially even probable, that updated legislation will fail to benefit community pharmacy practice and ultimately patients.

Future research could focus specifically on quantifying the time taken by pharmacies to process prior authorizations and complete step-therapy requirements, the financial impact of DIR fees, along with the number of prescriptions that are not picked up because patients cannot afford the copays. With this information in hand, clear guidance might be provided to legislators and lobbyists for how to not only increase the affordability of medications, but also ensure continued access to community pharmacists, who are critical members of the healthcare team.

## 5 Conclusion

This scoping review set out to evaluate the current literature examining the impact of PBMs on the finances of community pharmacies. The literature search revealed four articles, only one of which focused specifically on community pharmacy practice impacts. As legislation regulating PBMs continues to be considered by state governments across the country, additional research should be completed to ensure that these efforts have the best possibility of improving outcomes for patients by reducing costs and ensuring the viability of community pharmacy.

## Treatment of human subjects

IRB review/approval was not required.

## Declaration of Competing Interest

We declare no conflicts of interest or financial interests that the authors or members of their immediate families have in any product or service discussed in the manuscript, including grants (pending or received), employment, gifts, stock holdings or options, honoraria, consultancies, expert testimony, patents, and royalties.
